# Atypical presentation of cryptococcal meningitis in oncology patients: Case Report

**DOI:** 10.3389/fmed.2025.1661140

**Published:** 2025-10-21

**Authors:** Jing Wei, Haiyan Huang, Yingyu Zhang, Yanlan Huang, Yingfang Liang, Shan Jiang

**Affiliations:** ^1^Department of Neuro and Cardiovascular Medicine, Guangxi Medical University Cancer Hospital, Nanning, Guangxi, China; ^2^Department of Endocrinology and Metabolism Nephropathy, Guangxi Medical University Cancer Hospital, Nanning, Guangxi, China; ^3^Division of Molecular Signaling, Department of the Advanced Biomedical Research, Interdisciplinary Graduate School of Medicine, University of Yamanashi, Yamanashi, Japan

**Keywords:** cryptococcal meningitis, atypical clinical manifestations, Cancer, case report, clinical management

## Abstract

Cryptococcal meningitis (CM) is a serious central nervous system (CNS) infection primarily affecting immuno-compromised individuals, including cancer patients. Although rare in oncology populations, it may present atypically and pose diagnostic challenges. We report two cases of malignancy-associated CM with non-classical features. The first involved a male with lung squamous cell carcinoma and multiple comorbidities, presenting with mild cerebrospinal fluid (CSF) pleocytosis and normal opening pressure. The second case involved a female with chronic lymphocytic leukemia and concurrent cryptococcal and purulent meningitis, showing marked leukocytosis and elevated CSF pressure. Neither had recent antitumor therapy. Despite similar initial symptoms of fever and headache, their clinical courses diverged: the male recovered with antifungal treatment, while the female had a poor response and discontinued therapy. These cases highlight the variable and atypical nature of CM in cancer patients and underscore the importance of early recognition and inclusion of fungal infections in the differential diagnosis for CNS complications, even in the absence of classical features.

## Introduction

CM is a subacute or chronic meningoencephalitis caused by Cryptococcus species, and represents the most severe and frequent clinical manifestation of disseminated cryptococcal infection. Global surveillance estimates an annual incidence of approximately 250,000 cases, with over 181,000 associated deaths, highlighting its substantial global health burden (1).

As an opportunistic pathogen, Cryptococcus poses a significant risk to immunocompromised individuals. Human Immunodeficiency Virus (HIV)-associated immunosuppression is the predominant risk factor, accounting for up to 80% of cryptococcosis cases in Western countries. However, cases among non-HIV immunocompromised populations are increasingly recognized. These now represent approximately 5–10% of the total disease burden (2). Established risk factors in this group include prolonged corticosteroid or immunosuppressive therapy, solid organ transplantation, hematologic malignancies, and poorly controlled diabetes mellitus (3–5).

Among non-HIV patients, individuals with malignancies represent a particularly vulnerable subgroup. In these patients, CM often presents with atypical or non-specific clinical features, leading to frequent diagnostic delays and poorer outcomes (6, 7). The variability in presentation is likely influenced by the nature of the underlying malignancy (solid vs. hematologic), the degree of immune compromise, and concurrent comorbid conditions.

Here, we report two cases of CM in HIV-negative patients with distinct oncologic backgrounds. The first case involves a male patient with lung squamous cell carcinoma, diabetes mellitus, and membranous nephropathy, receiving long-term immunosuppressive therapy (tacrolimus and corticosteroids). The second case concerns a female patient with untreated chronic lymphocytic leukemia. Notably, neither patient had received recent antineoplastic treatment, yet both exhibited significant predisposing factors for invasive cryptococcal infection.

These cases highlight the diagnostic challenges posed by the variable manifestations of CM in oncology patients and emphasize the necessity for increased clinical vigilance. The divergent outcomes were likely influenced by factors including the type of underlying malignancy, host immune status, and therapeutic interventions.

## Case description

### Case 1

A 69-year-old male was admitted on August 30, 2023, with a two-month history of intermittent fever and headache. Before admission, the patient developed intermittent fever with peak temperatures up to 38.4 °C. The febrile episodes occurred irregularly in both frequency and duration, and were accompanied by chills and persistent distending headaches, most pronounced in the bilateral temporal regions. The patient denied vomiting, diplopia, vision loss, visual field deficits, limb numbness, or weakness. Headache intensity tended to diminish following defervescence, though it was associated with dizziness and blurred vision. Additionally, the patient reported an occasional paroxysmal cough productive of white mucoid sputum. Although antipyretic therapy provided temporary symptomatic relief, fever recurred repeatedly. The patient reported no contact with birds, avian droppings, or related materials prior to symptom onset, and no travel history to cryptococcus-endemic regions. His past medical history included stage II membranous nephropathy, type 2 diabetes mellitus, and hypertension. Renal function had stabilized following a course of corticosteroid and tacrolimus treatment. Diabetes and hypertension were controlled with metformin and nifedipine, respectively. Physical examination revealed stable vital signs and no lymphadenopathy. Cardiopulmonary and abdominal examinations were unremarkable. The patient was alert, oriented, and exhibited no behavioral or psychiatric abnormalities. Cognitive functions, including memory and calculation, remained intact. Cranial nerve examination was normal. Gait was steady without involuntary movements. Muscle tone and strength were normal (5/5) in all limbs, with intact sensation and physiological reflexes. Pathological reflexes were absent. Nuchal rigidity was present. Autonomic functions were normal.

**Figure 1:**
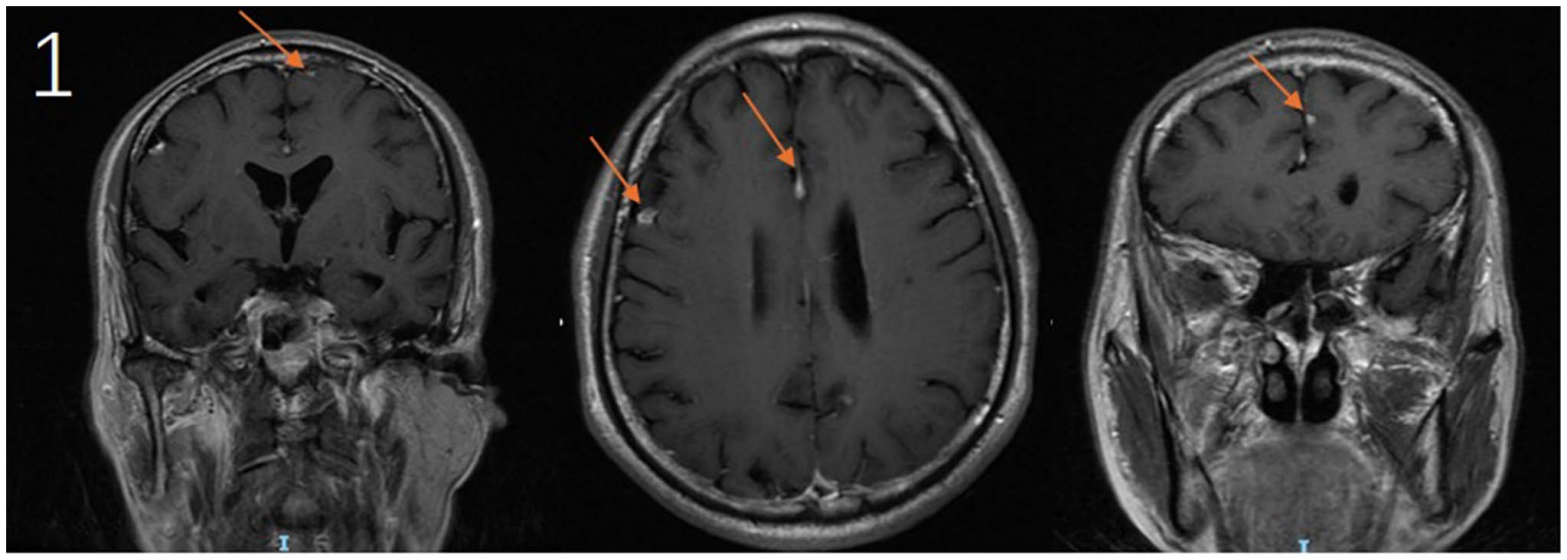
Cranial MRI demonstrates contrast-enhancing lesions in the following locations: the left frontal lobe, parafalcine region, right frontal lobe, and adjacent to the sulcus of the left parietal lobe (arrows).

Upon admission, the patient underwent a comprehensive diagnostic evaluation. Chest computed tomography (CT) identified a nodular lesion within the posterior segment of the right upper lobe. Cranial magnetic resonance imaging (MRI) demonstrated multiple contrast-enhancing lesions involving the left frontal lobe, parafalcine region, right frontal lobe, and the sulcus of the left parietal lobe (Figure 1). Laboratory investigations revealed elevations in the erythrocyte sedimentation rate (ESR) and high-sensitivity C-reactive protein (hs-CRP), while the white blood cell count, neutrophil percentage, and procalcitonin (PCT) level remained within normal limits. *Cryptococcus neoformans* capsular antigen test (CrAg) returned a positive result (Table 1). Additional testing, including autoantibody screening, (1,3)-*β*-D-glucan assay, Aspergillus galactomannan antigen testing, and HIV antigen/antibody assays, yielded negative results. No microbial growth was observed in cultures of bone marrow, blood, or bronchoalveolar lavage fluid. In light of the suspected malignant pulmonary nodules, the patient subsequently underwent bronchoscopy with biopsy. Histopathological examination of the endobronchial biopsy confirmed squamous cell carcinoma. In response to persistent headache and fever, a lumbar puncture was performed, which revealed an opening CSF pressure of 112 cm H₂O. CSF analysis demonstrated pleocytosis, elevated protein concentration, and hypoglycorrhachia. No malignant cells were identified on cytological examination (Table 2).

**Table 1:** Pre-treatment laboratory test results of blood.

Biochemical indicators	Reference range	Case 1	Case 2
Leukocytes (109/L)	3.5–9.5	7.16	25.7
Neutrophiles (109/L)	1.8–6.3	5.18	1.6
Lymphocytes (109/L)	1.1–3.2	1.12	23.21
Neutrophil percentage (%)	40–75	72.3	6.2
Monocytes (109/L)	0.1–0.6	0.69	0.90
ESR (mm/H)	0–15	62.2	-
hs-CRP (mg/L)	0–3	11	-
CRP (mg/L)	0–10	-	1.7
PCT (ng/mL)	0–0.05	0.037	0.036
CrAg	Negative	positive	positive

ESR, erythrocyte sedimentation rate; hs-CRP, high-sensitivity C-reactive protein; CRP, C-reactive protein; PCT, procalcitonin; CrAg, *Cryptococcus neoformans* capsular antigen test.

**Table 2:** Pre-treatment cerebrospinal fluid analysis.

CSF parameter	Reference Range	Case 1	Case 2
CSF pressure (mmH_2_O)	80–180	112	240
Appearance	Clear	Clear	Clear
Pandy test	Negative	Positive	Positive
CSF WBC (10^6^/L)	0–5	317	1,100
CSF N (%)		21.2	0.2
CSF L (%)		78.7	99.7
CSF E (%)		0.1	0.1
CSF protein (g/L)	0.08–0.45	1.98	3.58
CSFchloride ion (mmol/L)	120–130	116	110
CSF glucose (mmol/L)	2.5–4.4	1.9	1.05
Tuberculosis smear of CSF	Negative	Negative	Negative
CSF Routine Smear for Bacteria	Negative	Negative	Negative
CSF India Ink Test	Negative	Negative	Positive
mNGS	Negative	-	Positive
CSF-ECE		Abundant inflammatory cells	Numerous small lymphocytes

CSF, cerebrospinal fluid; CSF WBC, cerebrospinal fluid white blood cell; CSF N, cerebrospinal fluid neutrophils; CSF L, cerebrospinal fluid lymphocytes; CSF E, cerebrospinal fluid eosinophilic; CSF India Ink Test, india Ink Preparation of cerebrospinal fluid for Cryptococcus; mNGS, metagenomic next-generation sequencing technology; CSF-ECE, Cerebrospinal Fluid Exfoliative Cytology Examination.

Integrating these diagnostic results with the patient’s medical history confirmed the following conditions: CM, squamous cell carcinoma of the right upper lobe, type 2 diabetes mellitus, and hypertension. Treatment for CM was initiated with daily administration of amphotericin B cholesterol complex (280 mg) and flucytosine (1.5 g every 6 h) from September 2 to September 12, 2023. A repeat lumbar puncture on September 4 was intended to evaluate for autoimmune encephalitis and to perform CSF metagenomic next-generation sequencing (mNGS) for exclusion of other infections; however, due to financial constraints, only mNGS was conducted. The opening pressure was 152 cmH₂O. Both CSF culture and mNGS (the relative abundance of *Cryptococcus neoformans* was 20.45%) confirmed *Cryptococcus neoformans* infection, with no other pathogens detected. CSF analysis at this time showed persistent pleocytosis (387 × 10^6^/L), a mildly elevated protein level (2.37 g/L), and decreased glucose (1.88 mmol/L) and chloride (106 mmol/L) concentrations. On September 8, PCT was mildly elevated (0.069 ng/mL), while hs-CRP remained stable (11.81 mg/L). After treatment, the patient’s fever and headache gradually resolved with no deterioration in neurological function. Given the active fungal infection, systemic antitumor therapy was deferred. The patient was discharged in stable condition following clinical improvement and resolution of the infectious symptoms.

Following discharge, the patient was referred to a local hospital and completed an additional month of antifungal therapy consisting of daily amphotericin B cholesterol complex (280 mg) and flucytosine (1.5 g every 6 h). The patient returned for an outpatient follow-up visit upon completion of this treatment and reported complete resolution of both headache and fever. Although hospitalization was recommended for further management of his lung tumor, the patient declined admission. There has been no further contact with him since that visit, and all subsequent attempts to reach the patient by telephone have been unsuccessful.

### Case 2

On November 25, 2024, a 67-year-old female was admitted to the Department of Hematological Oncology and Pediatric Oncology with a chief complaint of recurrent fever and headache persisting for more than 10 days. Her symptoms began on November 16, 2024, with intermittent fever reaching a maximum temperature of 38.5 °C, accompanied by a persistent headache. Additional symptoms included altered mental status, generalized fatigue, reduced appetite, and sleep disturbances. Although antipyretic medications provided temporary relief, the fever recurred despite oral antibiotic therapy, leading to hospitalization. The patient reported no known exposure to birds, bird droppings, or related materials, and no travel history to cryptococcus-endemic regions prior to symptom onset. The patient had a known history of chronic lymphocytic leukemia/small lymphocytic lymphoma (CLL/SLL). As her condition did not meet the criteria for treatment initiation, no therapeutic intervention had been administered. Over the preceding 3 months, she experienced multiple recurrent episodes of herpes zoster infection. There was no history of diabetes mellitus, hypertension, or other significant comorbidities, and no underlying immune disorders were documented. Her physical exam revealed that she was in stable condition but exhibited a depressed mental state, appearing quiet and withdrawn. Multiple enlarged lymph nodes were palpated bilaterally in the cervical regions. The largest node measured approximately 3 × 3 cm, with a firm consistency, fixed position, no tenderness, and an intact overlying skin without ulceration. Cardiopulmonary and abdominal exams were unremarkable. Cognitive functions, including orientation, memory, and calculation, remained intact. No abnormalities were detected on cranial nerve examination. The patient’s gait is normal, with no involuntary movements observed. Muscle tone and strength in all limbs are normal (grade 5/5). Both deep and superficial sensations are intact. Deep tendon reflexes and superficial reflexes are physiological, and no pathological reflexes are present. Nuchal rigidity was noted; however, coordination remains unimpaired. Autonomic function appears normal.

Following admission, laboratory investigations revealed leukocytosis with a normal neutrophil percentage. Inflammatory markers, including C-reactive protein (CRP) and PCT, were within normal limits (Table 1). Serological testing for HIV antigen/antibody was negative. Given the presentation of recurrent fever and headache, along with a history of recurrent herpes zoster infections over the past 3 months, viral encephalitis or meningitis was suspected. Empirical anti-infective therapy was initiated with cefoperazone-sulbactam (3 g every 12 h), moxifloxacin (0.4 g daily), and acyclovir (0.5 g every 8 h). Despite this treatment, the patient remained febrile. Due to increasing concern for intracranial infection, she was transferred to our unit on November 26, 2024.

**Figure 2:**
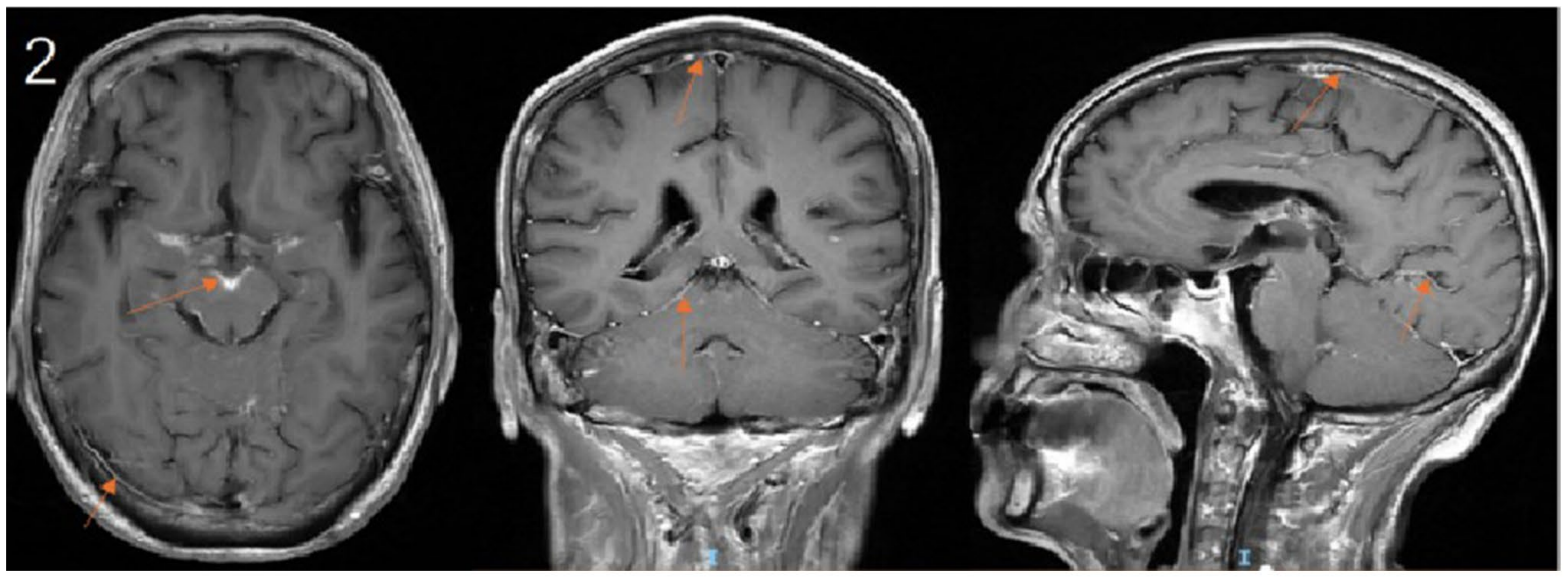
Contrast-enhanced cranial MRI demonstrates leptomeningeal enhancement along the sulci and cisterns. (arrows).

Upon transfer to our department, the existing anti-infective regimen was maintained, and a comprehensive diagnostic workup was initiated. Cranial MRI demonstrated multiple areas of leptomeningeal enhancement (Figure 2). Lumbar puncture revealed an elevated opening pressure of 240 mmH₂O. CSF analysis showed marked pleocytosis, elevated protein, and decreased glucose and chloride levels. Cytological examination identified abundant small lymphocytes, suggestive of CNS involvement by lymphoma (Table 2). mNGS of the CSF detected microbial genomes corresponding to *Cryptococcus neoformans* (77.78% relative abundance) and *Pseudomonas aeruginosa* (28.57% relative abundance). No pathogenic autoantibodies associated with autoimmune encephalitis were detected. Additionally, CSF smear confirmed the presence of *Cryptococcus neoformans*, and the CrAg was positive.

**Figure 3:**
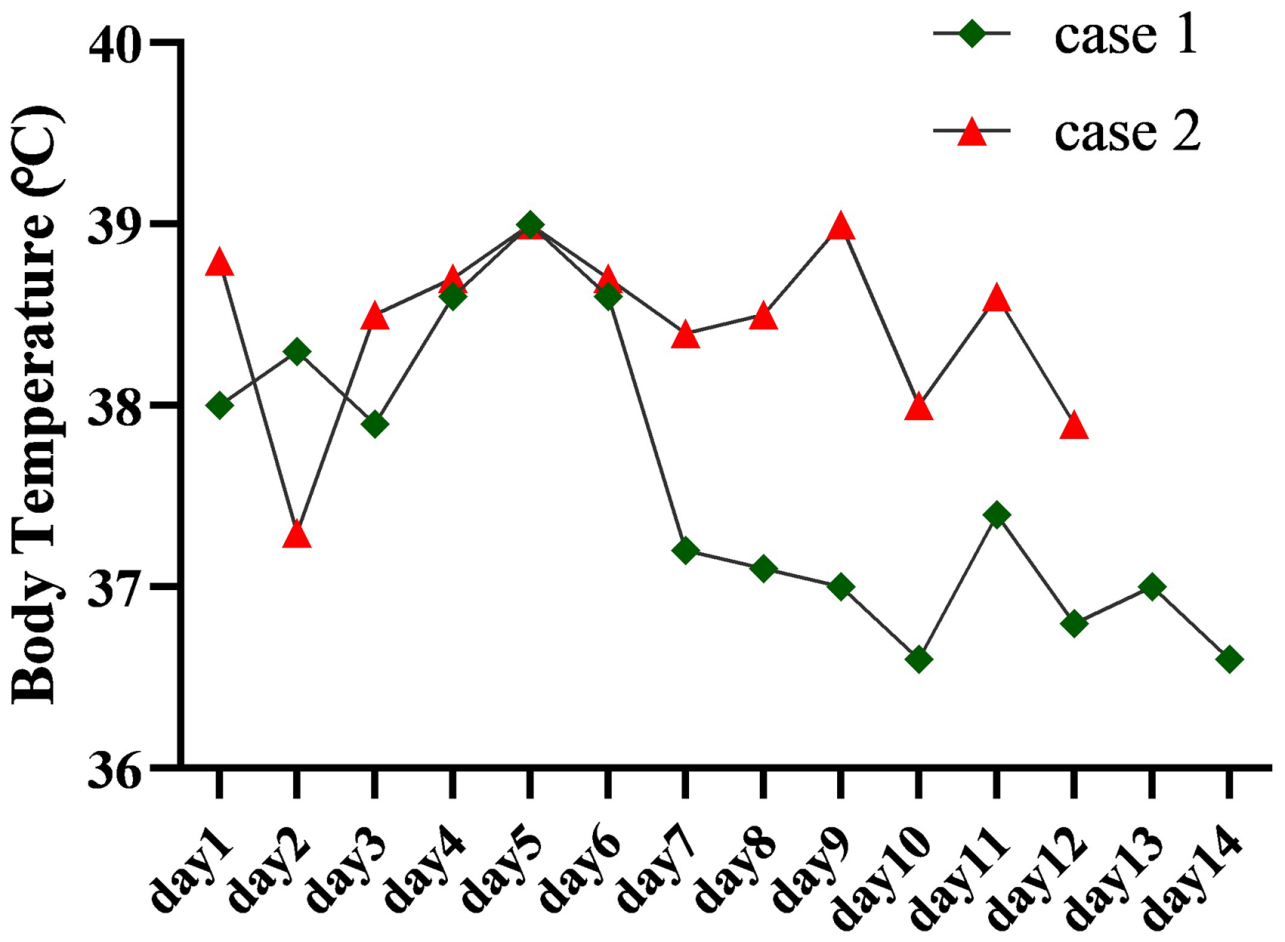
The figure traces the body temperature profiles of two patients throughout their hospitalization. Case 1 exhibited fever resolution following treatment, whereas the fever in Case 2 proved refractory and persisted.

Based on these collective findings, the patient was diagnosed with concurrent cryptococcal and bacterial meningitis. The recommended initial antimicrobial therapy included amphotericin B, flucytosine, and piperacillin-tazobactam. However, due to concerns regarding the cost and potential toxicity of amphotericin B, the patient declined this treatment. On November 28, 2024, the antifungal regimen was adjusted to intravenous fluconazole (0.4 g every 12 h) in combination with piperacillin-sulbactam sodium (3 g every 6 h). Following initiation of the modified antimicrobial therapy, the patient continued to experience intermittent fever. On December 2, 2024, after consultation with the clinical pharmacy team, the antimicrobial regimen was escalated to meropenem (2 g every 12 h) combined with fluconazole (0.4 g every 12 h). Follow-up laboratory testing on December 5, 2024, revealed a decrease in white blood cell count but an increase in CRP levels compared to previous results. Despite these adjustments, the patient remained febrile (Figure 3) and developed drowsiness. Neurological examination revealed no significant changes, indicating a poor therapeutic response and an unfavorable prognosis.

After a thorough discussion of the clinical prognosis, the patient and her family elected to discontinue treatment and were discharged against medical advice. The patient subsequently sought care at another hospital but was soon discharged again due to the poor prognosis. All subsequent attempts to reestablish contact were unsuccessful, and no further follow-up could be conducted.

## Discussion

Cryptococcosis is a classic opportunistic infection, primarily affecting immuno-compromised individuals. In oncology patients, several factors contribute to immune dysfunction, including tumor-induced disruption of immune surveillance mechanisms and immunosuppression mediated by the tumor microenvironment. These pathophysiological changes create a profoundly immunodeficient state, significantly increasing susceptibility to opportunistic infections such as cryptococcal disease (8). Among HIV-negative individuals, the most significant risk factors for cryptococcosis include malignancies, sarcoidosis, chronic corticosteroid therapy, tacrolimus use, and diabetes mellitus (3–5).

Tumor-associated CM often presents with attenuated CSF inflammatory response and atypical clinical features. Notably, only 5% of cancer patients with concurrent meningitis exhibit the classic triad of fever, neck stiffness, and altered mental status (6). In one of the two cases we reported, a male patient with lung squamous cell carcinoma presented with an atypical clinical course. Prior to his cancer diagnosis, he had comorbid diabetes mellitus and membranous nephropathy, for which he had received long-term corticosteroid and tacrolimus therapy. His presentation deviated from typical CM cases, as the lumbar puncture revealed normal CSF opening pressure—a rare finding that can complicate diagnosis. Similar cases have been documented in the literature, including instances where CM in non-cancer patients was initially misdiagnosed as normal pressure hydrocephalus (9–11). The patient’s underlying conditions, including membranous nephropathy and diabetes mellitus, not only predisposed him to cryptococcal infection but also contributed to the diagnostic complexity.

CM frequently manifests with nonspecific symptoms, which can obscure timely diagnosis and lead to delays in treatment (5). In this case, the patient presented with fever and headache as primary complaints. Neuroimaging revealed multiple contrast-enhancing cerebral nodules, which, in the context of his history of lung squamous cell carcinoma, raised strong suspicion for metastatic intracranial disease. This posed a significant diagnostic challenge. Ultimately, the diagnosis of CM was confirmed through comprehensive CSF analysis, including *Cryptococcus neoformans* smear and culture. This case highlights the critical importance of thorough CSF evaluation in immunocompromised patients, as reliance on radiographic findings alone may lead to diagnostic delays. Such delays, coupled with suboptimal antimicrobial therapy, are well-established contributors to adverse clinical outcomes in cryptococcal CNS infections (12). In immunocompromised cancer patients presenting with fever and headache, routine CSF CrAg should be performed regardless of CSF pressure measurements, given their heightened susceptibility to opportunistic infections. The second case involves a female patient with CLL who was hospitalized for recurrent fever and headache. Unlike the first case, this patient exhibited marked leukocytosis in both peripheral blood and CSF, accompanied by significantly elevated CSF opening pressure (240 mmH₂O). Such findings deviate from the typical presentation of CM, which is usually characterized by normal or mildly elevated leukocyte counts (13). These atypical features could have been easily misinterpreted as manifestations of her underlying CLL, potentially delaying diagnosis. Initial clinical suspicion of bacterial co-infection was later confirmed by CSF mNGS, which identified dual infections with *Cryptococcus neoformans* and *Pseudomonas aeruginosa*. Despite multiple adjustments to her antimicrobial regimen targeting both pathogens, the patient continued to experience recurrent fever and headache. Ultimately, the patient and her family chose to discontinue treatment and requested discharge against medical advice.

Therefore, in patients with malignant tumors who develop neurological symptoms, such as fever and headache, multiple etiologies should be considered. The primary diagnostic goals are to determine whether the patient has intracranial metastasis, autoimmune encephalitis, or an intracranial infection. Single or multiple pathogens may cause intracranial infections. Thus, we recommend the following tests for differential diagnosis: contrast-enhanced MRI of the head, CSF cytology, CSF antibody testing for autoimmune encephalitis, CSF mNGS, and CrAg.

The two cases illustrate distinct clinical outcomes: the female patient with CLL demonstrated poor treatment response, while the male patient with lung squamous cell carcinoma achieved favorable therapeutic results. Several factors may explain this disparity. Currently, no universally established treatment standard exists for HIV-negative patients with CM. Multiple studies and clinical guidelines recommend amphotericin B in combination with flucytosine as the first-line induction therapy. Although some reports have documented the use of amphotericin B alone or fluconazole monotherapy, the combination of amphotericin B and flucytosine has been associated with superior survival outcomes in non-HIV-infected individuals (7, 8, 14–16). For CM, our team employs consistent anti-infection regimens for both tumor and non-tumor patients. The principal distinction lies in the duration of induction therapy, which is tailored individually for cancer patients based on specific clinical factors. These include tumor type, rate of progression, and the patient’s overall tolerance to treatment. In cases with a high risk of rapid tumor progression, the combined regimen of amphotericin B and flucytosine is abbreviated to approximately 2 weeks, followed by maintenance therapy with oral fluconazole. Additionally, the patient’s financial capacity is carefully considered when designing the antifungal treatment plan. Throughout subsequent combined antitumor and antifungal therapy, our team conducts close monitoring of blood counts, liver function, and renal function. However, the CLL patient received fluconazole monotherapy due to financial constraints and concerns about amphotericin B toxicity, especially in the context of hematologic malignancy. In contrast, the lung cancer patient adhered to the recommended treatment regimen. Although fluconazole is effective against CM (17), the choice of regimen may influence prognosis, warranting further investigation. Notably, susceptibility testing in the male patient revealed intermediate sensitivity to fluconazole, while no CSF cultures were obtained for the CLL patient, leaving the possibility of fluconazole resistance unexamined—a potential contributor to treatment failure. Meanwhile, the CLL patient faced additional complications, including concurrent *Pseudomonas aeruginosa* meningitis and suspected CNS lymphoma infiltration. These factors likely compounded the therapeutic challenges and increased the risk of poor outcomes. Furthermore, studies suggest that involvement of arachnoid granulations, where abundant cryptococcal cells often accumulate, correlates with elevated CSF pressure in CM (18). The significantly higher CSF pressure observed in the CLL patient compared to the male patient may reflect a greater fungal burden, potentially contributing to her worse prognosis. Based on our team’s clinical experience, we have observed that the prognosis of CM in this patient population appears to be influenced by both polymicrobial co-infection and intracranial metastasis, despite considerable variability in clinical presentation. These findings, though derived from a limited two-case series, highlight the need for larger multicenter studies to further elucidate the complexity of this condition.

## Conclusion

CM in patients with malignancies often exhibits non-specific clinical manifestations. Therefore, routine CrAg in CSF is strongly recommended for those with persistent fever or headache, even when typical risk factors are absent. Due to the high prevalence of polymicrobial co-infections in this population, comprehensive diagnostic approaches, including mNGS and antifungal susceptibility testing, are essential to guide targeted and effective treatment.

## Data Availability

The original contributions presented in the study are included in the article/supplementary material, further inquiries can be directed to the corresponding author.
